# Structure and formation of copper cluster ions in multiply charged He nanodroplets†

**DOI:** 10.1039/d2cp04569a

**Published:** 2023-03-14

**Authors:** O. V. Lushchikova, M. Gatchell, J. Reichegger, S. Kollotzek, F. Zappa, M. Mahmoodi-Darian, P. Scheier

**Affiliations:** a Institut für Ionenphysik und Angewandte Physik, Universität Innsbruck Technikerstr. 25 A-6020 Innsbruck Austria Olga.Lushchikova@uibk.ac.at; b Department of Physics, Stockholm University SE-10691 Stockholm Sweden; c Department of Physics, Karaj Branch, Islamic Azad University Karaj Iran

## Abstract

The structure of cationic and anionic Cu clusters grown in multiply charged superfluid He nanodroplets was investigated using He tagging as a chemical probe. Further, the structure assignment was done based on the magic-numbered ions, representing the most energetically favorable structures. The exact geometry of the cluster and positions of He is verified by calculations. It was found that the structure of the clusters grown in the He droplets is similar to that produced with a laser ablation source and the lowest energy structures predicted by theoretical investigations. The only difference is the structure of the Cu_5_^+^, which in our experiments has a twisted-X geometry, rather than a bipyramid or planar half-wheel geometry suggested by previous studies. This might be attributed to the different cluster formation mechanisms, the absence of the Ar-tag and the ultracold environment. It was also found that He tends to bind to partially more electro-negative or positive areas of the anionic or cationic clusters, respectively.

## Abstract 2

The most stable structure of cationic (left) and anionic (right) Cu_5_ was determined by particularly stable configurations with He.

## Graphical abstract

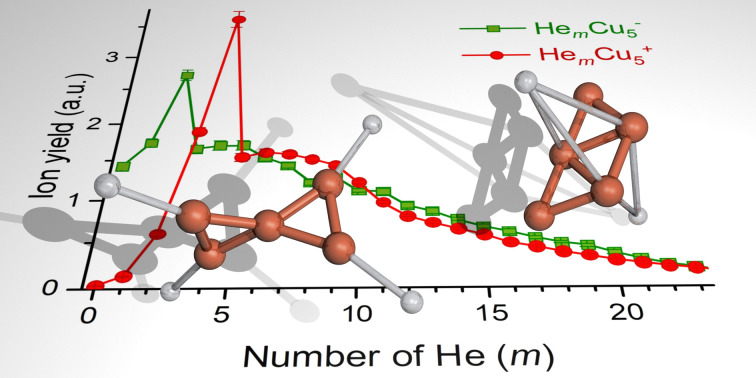

## Introduction

In the past decades, the investigation of metal clusters has been motivated by various fields such as catalysis, nanomaterials, and solid-state physics. The chemical and physical properties of metal clusters are quite different from both atom and bulk and exhibit for small clusters a non-linear dependence on the cluster size. Moreover, they have a high surface-to-volume ratio, which makes them especially interesting for the design of catalytic materials.^1^ Recently, Cu clusters have attracted a lot of attention. They are considered to be promising candidates for the catalytic hydrogenation of CO_2_ to methanol. It was experimentally and theoretically shown that small Cu clusters deposited on a surface have higher catalytic activity than a conventional Cu-based catalyst.^2–4^ It has been shown that the reactivity of a copper cluster is strongly dependent on its geometrical and electronic structure.

Cu clusters were among the first metal clusters produced.^5^ In the past decades, many studies were focused on the characterization of these clusters in different charge states by investigating even–odd oscillations of the ion yield as a function of the cluster size,^6,7^ electronic shell closures,^6,8,9^ ionization energies,^8^ electron affinities,^10,11^ electric dipole polarizabilities^12^ and so on. Since the interest in metal clusters was growing and computational techniques were developing, gas-phase Cu clusters became a good role model for theoretical investigations.^13–17^ Copper atoms, similarly to silver and gold, have a rather “simple” electronic configuration [Ar]3d^10^4s^1^, with a closed d shell and a single electron in an s orbital, which strongly determines the activity of coinage metal clusters.

More recently, the structure of Cu_*n*_^+^ clusters, with *n* = 3–10, has been determined by means of messenger spectroscopy using IR light in the fingerprint region (70–280 cm^−1^).^18^ This study was complemented by density functional theory (DFT) calculations and the influence of Ar as a messenger was investigated in detail.^19^ Another study has determined the structure of the Cu_*n*_^+^ in the same size range with ion mobility mass spectrometry.^20^ The experimental results were also interpreted with DFT calculations. In general, both of these experimental studies are in good agreement. However, there is some controversy considering the structure of Cu_5_^+^, whether it is three-dimensional (3D) or planar (2D). This difference might be developed during the cluster formation or caused by the addition of the Ar-messenger. The structure of neutral Cu clusters in Ar shells was also studied using X-ray absorption near-edge spectroscopy, however, this study was mainly focused on the neutral Cu_13_.^21^

In the present study, we utilize a complementary method to investigate the structure of small copper clusters, with special emphasis on Cu_5_^+^. Recently, the well-known approach for cluster formation within superfluid He nanodroplets (HNDs)^22^ has been improved, allowing pickup into multiply charged HNDs.^23^ Unlike in neutral HNDs, charges act as nucleation centers for cluster formation attracting dopant atoms *via* charge-induced dipole interaction. Multiple clusters can be grown within one charged HND, resulting in increased signal intensity and a decreased width of the cluster size distribution.^24^ Due to the ultracold environment of the He droplets, it is possible to obtain hints on the structure of the Cu_*n*_^+^ clusters using He-tagging as a probing method.^25^ For this, the abundance of the formed He_*m*_Cu_*n*_^+^ complexes is investigated with high-resolution mass spectrometry.

Cu_*n*_^+^ clusters, with *n* < 8, were formed upon electron ionization of neutral HNDs doped with Cu.^26^ An odd–even oscillation of the ion yield was attributed to the electronic shell structure. These oscillations are also typical for other coinage^27,28^ or alkali metals^29–31^ that have similar properties due to their electronic configuration. The solvation of Cu^+^ and Cu_2_^+^ formed in neutral He droplets was reported previously by our group.^32^ It was found that Cu^+^ solvated with 6, 12 and 24 He atoms have the most stable structures, while for *n* = 2 the most stable structure is He_2_Cu_2_^+^. The combination of the two copper isotopes as well as the pickup of residual water and the low intensity of He-tagged ions made the assignment of He-solvated copper cluster ions challenging. Unlike copper, gold is monoisotopic and a higher amount of He-tagged gold cluster ions could be produced. This enabled the identification of He_*m*_Au_*n*_^+^ complexes in mass spectra for *n* up to 14 already in neutral He-nanodroplets.^33^ From the relative abundances of the He solvated gold cluster cations and complimentary DFT calculations the cluster structures of Au_*n*_^+^ clusters could be determined. Although the formation of metastable structures has been reported in the ultracold environment of the HNDs,^25^ it was concluded that only ground state gold structures are formed within the droplets, in good agreement with the literature. Tiefenthaler *et al.* recently reported that high yields of He-tagged ions can be achieved upon pickup into multiply charged HNDs and subsequent liberation of the ions from the host droplet by multiple collisions with room temperature He gas.^23^ The additional use of isotope enriched ^63^Cu enables the unambiguous assignment of He_*m*_Cu_*n*_^+^ with *n* up to 10 and *m* up to 100. Moreover, with the present technique also He-tagged anionic clusters are efficiently formed.^34^ Therefore, in this paper, for the first time, He attachment is used for the structural characterization of anionic metal clusters (Cu_*n*_^−^, with *n* = 2–6).

## Methods

### Experimental

The experimental instrument was described in detail elsewhere.^23^ In short, superfluid He droplets are produced in a supersonic expansion of pre-cooled He gas (99.9999% ^4^He) at pressures between 22 and 31 bar and temperatures between 9.1 and 9.5 K through a pinhole nozzle with a diameter of 5 μm. In the case of cations, neutral He droplets with an average size of about 10^6^ are ionized by electron ionization with an electron current of 350 μA and energy of 57 eV. For anions, we expanded 31 bar He at a temperature of 9.1 K and set the electron energy to 22 eV at an electron current of 230 μA. Further, the charged droplets are mass to charge selected by passing through an electrostatic quadrupole bender and doped with monoisotopic copper (99.9% ^63^Cu) in the pickup chamber. The Cu vapor is formed by heating small copper slices in an oven, made of shapal ceramic, which is heated up to 1050 K (measured on the outside surface of the oven by a thermocouple) by 130 W. The temperature inside the oven exceeds the melting temperature of the copper of 1358 K,^35^ which is indicated by the formation of a copper sphere upon melting the metal slices. Copper atoms picked up by the HND are polarized and attracted by the charge centers located inside (in the case of singly charged HNDs) or close to the surface (in the case of multiply charged HNDs) of the HNDs. Charge transfer or electron attachment ionizes the first copper atom colliding with an initial charge center (He_3_^+^ solvated by additional He atoms and He*^−^ in the case of positively^36^ and negatively^37^ charged HNDs, respectively) and the attachment of additional copper atoms forms singly charged copper clusters. These cluster ions are then liberated from the He droplet in the evaporation chamber, *via* collisions with room temperature He gas. Shrinking of the HNDs reduces the distance between the embedded copper cluster ions and leads to sequential ejection of charge centers still solvated with a few 100 He atoms.^24^ Additional collisions of these ions with He atoms remove gradually the He solvation layer. The amount of He atoms attached to the cluster ions is determined by the pressure of the He gas which is introduced to the chamber for evaporation of the host droplets. Higher pressure leads to more collisions and, therefore, to fewer He atoms attached to the copper cluster ions. Finally, He_*m*_Cu_*n*_^+^ complexes are detected in a time-of-flight mass spectrometer.

The obtained spectra are analyzed using the in-house software called IsotopeFit.^38^ This package allows for calibration, background correction and automatic fitting of the mass spectra. In this way, even difficult overlapping contributions from different isobaric ions can be deconvoluted.

### Theory

The experimental results in this work are supported by electronic structure calculations of charged Cu clusters solvated by He. The bare copper structures were selected based on the previous computational studies^13–17^ and optimized using second order Møller–Plesset perturbation theory (MP2) after test calculations showed that this method gives results comparable to significantly more expensive coupled cluster methods, both in terms of structures and interaction energies (see ESI† for additional details). The triple-zeta Karlsruhe basis set with diffuse functions, def2-TZVPD, was used for all of the calculations. All of the calculations were carried out using the Gaussian 16 software.^39^ Due to the weak interaction between He and the Cu clusters, basis set superposition errors (BSSEs) can be significant (on the same order of magnitude as the binding energies). We have therefore optimized the He-tagged clusters on a counterpoise-corrected potential energy surface. This correction is also used when calculating vibrational frequencies for identifying real energy minima as well as for determining zero-point corrections to the binding energies. Harmonic zero-point energy corrections were calculated to confirm actual energy minima, but these corrections are only included in reported energies when explicitly noted.

## Results and discussion

**Fig. 1:**
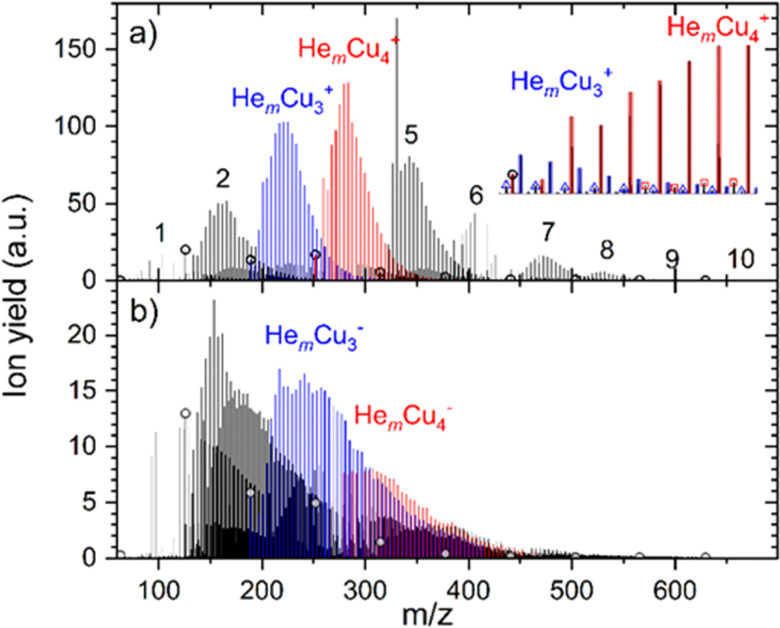
(a) Mass spectrum showing He_*m*_Cu_*n*_^+^ ions extracted from multiply charged HNDs doped with ^63^Cu *via* multiply collisions with He gas at a pressure of 9 × 10^−4^ mbar. The number *n* of Cu atoms in the ions is indicated by the numbers on top of the pronounced peak series. Pristine copper cluster ions (*m* = 0 He atoms) are indicated by black open circles. For *n* = 3 and 4, the He_*m*_Cu_*n*_^+^ peaks are designated by blue and red bars, respectively. The inset shows a smaller section and two minor peak series due to a small water impurity are assigned to He_*m*_H_2_OCu_3_^+^ (blue open triangles) and He_*m*_H_2_OCu_4_^+^ (red open squares). (b) Mass spectrum of anions obtained by doping negatively charged HNDs with copper vapor. The density of the copper vapor and the He pressure in the evaporation cell were identical to (a), however, the number of He atoms per charge selected in the quadrupole bender was 1.86 × 10^5^ instead of 3.31 × 10^5^ in the case of cations. Please note that the peaks in the range of *n* = 1 are dominated by He_*m*_H_2_OCu^−^.

The He adsorption on Cu_*n*_^+^ (*n* = 1–10) is investigated by liberating He_*m*_Cu_*n*_^+^ complexes from the superfluid He droplet hosts. A typical mass spectrum obtained for the lowest studied evaporation pressure (most He attached) is illustrated in Fig. 1. In the case of cations, shown in Fig. 1a, we selected multiply charged HNDs containing 3.31 × 10^5^ He atoms per charge and the mass spectrum is dominated by He-tagged copper cluster ions He_*m*_Cu_*n*_^+^, with *m* up to 100 and *n* up to 10. Pristine copper cluster ions are designated by open circles and are under these conditions less abundant than He solvated copper cluster ions. Mass peaks due to the pickup of water from the residual gas and a stated impurity of 1 ppm in the He gas used to evaporate the HNDs in the RF-hexapole are small and do not compromise the He-tagged copper cluster ion series. Ions that contain one H_2_O molecule are visible, as indicated by the open triangles and squares in the inset of Fig. 1a, but they are more than an order of magnitude less abundant than water-free He_*m*_Cu_*n*_^+^ ions.

For anions, shown in Fig. 1b, water contamination is more problematic. For Cu^−^ the dominant peak series is He_*m*_H_2_OCu^−^, consuming all anionic copper monomers. Therefore, the signal intensity on He_*m*_Cu^−^ is too low to be analyzed. Also, He_*m*_H_2_OCu_2_^−^ is almost as abundant as the water-free He-tagged copper dimer anion. With an increasing copper cluster size, the water contamination gets less severe. Anionic HNDs have to contain more than 4 million He atoms in order to carry two negative charges^34^ whereas the critical size for cationic doubly charged HNDs is only 10^5^.^40^ We selected anionic HNDs that contain 1.86 × 10^5^ He atoms per charge and these are exclusively singly charged. Thus, the complete surface of the HNDs is the cross-section for the pickup of dopants to this single charge center. In contrast, the average charge state of the cationic HNDs was three allowing larger droplets to pass the quadrupole bender, however higher charge state reduces the available pickup cross-section per charge center. If the same number of He atoms per charge for anions and cations is selected, a charge state of +10 reduces the effective pickup cross-section per charge center to 46% and for a charge state of +100 to 21%. The cross-section for pickup does not depend on the charge state and polarity of HNDs. In multiply charged HNDs the dopants will be distributed among the charge centers, which again results in a cluster size distribution that follows the Poisson statistics. In contrast, all dopants are forming one large cluster in a singly charged HND. However, the more important factor for the reduction of water contamination for cations in this experiment is the evaporation of He atoms in the RF-hexapole. In the case of anions, almost the complete HNDs have to be vaporized which requires an enormous amount of collisions, whereas in the case of multiply charged cations, a partial evaporation already leads to the ejection of charge centers as soon as the critical size for the given charge state is reached.

### Cationic clusters

For cations, we have investigated twelve different pressure regimes in total, starting with only one He atom attached to Cu_*n*_^+^ at 3 × 10^−3^ mbar pressure, increasing to over 80 He at 7 × 10^−4^ mbar. The ion yields of He_*m*_Cu_*n*_^+^ are plotted as a function of *m* for different *n* at all pressure regimes in Fig. S1, which can be found in ESI.† Since the attachment of He at higher pressures (up to 3 × 10^−3^ mbar) follows exponential distributions and shifts to lognormal by lowering the evaporation pressure, here we will present only pressure regimes in the lower pressure range, 9 × 10^−4^ and 7 × 10^−4^ mbar, called high and low pressure through the text for simplicity. For these pressures, the intensity of He_*m*_Cu_*n*_^+^ is particularly high for *m* in the range where Goulart *et al.* reported magic numbers and shell closures for He_*m*_Au_*n*_^+^.^33^ Moreover, to ensure that these magic number ions do not depend on the conditions, we have repeated the experiment at 7 × 10^−4^ mbar pressure. All the conditions during this experiment were different, besides the pressure in the collision cell. The comparison between the two settings is described in Table S1 and Fig. S2 (ESI†). Although the overall cluster distribution shifted to larger clusters and there is slightly more He attached, we have concluded that the overall pattern and magic number ions are not influenced.

**Fig. 2:**
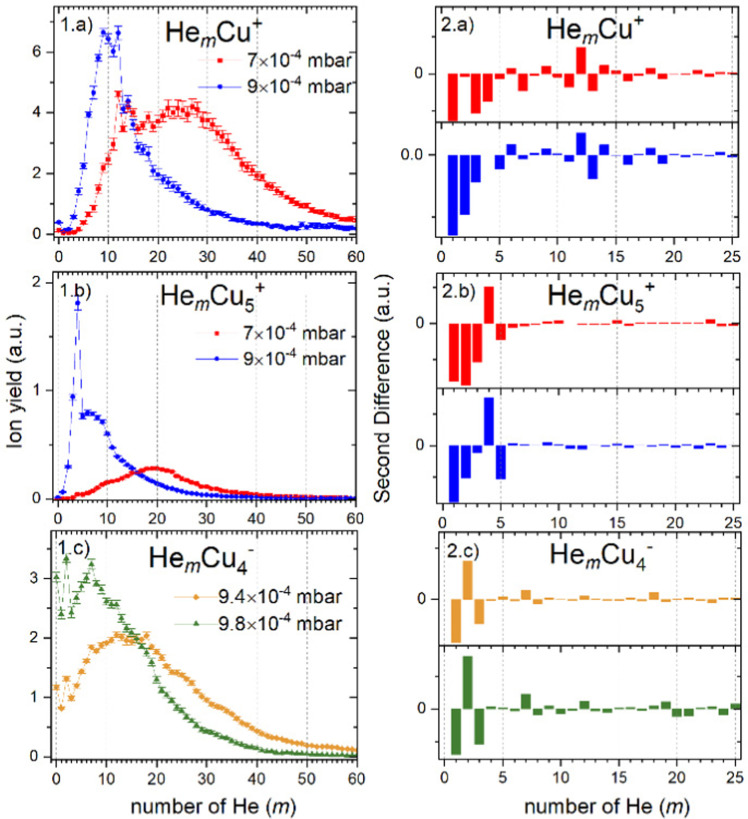
Left: The cluster size distributions of selected He_*m*_Cu_*n*_^+/−^. Plots (1.a) and (1.b) show the cations measured at 7 × 10^−4^ (red squares) and 9 × 10^−4^ mbar (blue circles). Plot (1.c) shows the distributions of anions measured at 9.4 × 10^−4^ (orange diamonds) and 9.8 × 10^−4^ (green triangles). The symbols represent the ion yield of every *m* and are complemented with an error bar. Right: The second difference of the selected cluster size distributions of He_*m*_Cu_*n*_^+/−^. Plots (2.a) and (2.b) show the cations measured at 7 × 10^−4^ (red bars) and 9 × 10^−4^ mbar (blue bars). Plot (2.c) shows the distributions of anions measured at 9.4 × 10^−4^ (orange bars) and 9.8 × 10^−4^ (green bars) mbar.

The He distributions measured for different cluster sizes are presented in Fig. 2 (left) for selected cluster sizes. The figure describing the whole set of measured cluster sizes can be found in ESI† (Fig. S3). However, due to a high number of data points, it is easy to overlook some magic numbered ions. Therefore, for each distribution also the second difference is calculated and shown in Fig. 2 (right), the full overview is in Fig. S4 (ESI†). This method considers the logarithm of the intensity difference of an ion to the two neighboring cluster ions. In this way, the outlying data points can be identified with higher precision. To calculate the second difference following formula is used:
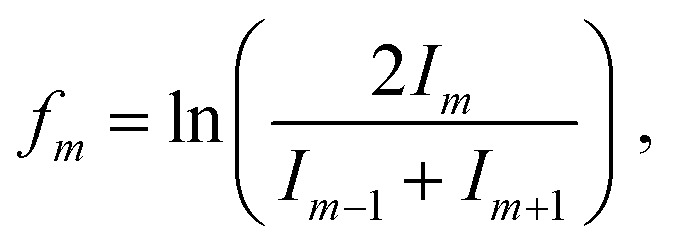
where *I*_*m*_ is the ion intensity of complexes with *m* He atoms. Although in some cases many magic numbers have been found with this method, here, we discuss only the most outstanding. The list of assigned magic numbers can be also found in Table 1. Among all studied Cu_*n*_^+^ ions, a single Cu^+^ ion binds the most He atoms, with *m* up to 100. The ion yield distribution of He_*m*_Cu^+^ complexes, shown in Fig. 2.1.a, is quite rich. In the pressure regime illustrated in Fig. 1, there is nearly no bare Cu^+^ left. Also, *m* = 1 and 2 are absent. The distribution starts at *m* = 3 and 4 for the measurements performed at high (9 × 10^−4^ mbar) and low (7 × 10^−4^ mbar) evaporation pressures, respectively. The most obvious magic number ions at *m* = 6, 9, 12 (overall maximum), 14, 18 and 24 are visible in both pressure regimes.

**Table 1:** Summary of all assigned magic numbers based on Fig. 2 and 3. Magic numbers assigned in previous work^32^ for He_*m*_Cu^+^ and He_*m*_Cu_2_^+^ are underlined. An approximate maximum of the He atoms attached to each cluster size is also reported

*n*	Max, *m*	Magic, *m*
Cations		
1	100	6̲, 9, 1̲2̲, 14, 18, 22, 2̲4̲
2	80	2̲, 6, 12, 17, 26
3	70	3, 6, 18, 20, 27
4	60	2, 4, 8, 24
5	50	4, 9
6	50	2, 4, 9
7	50	2, 7, 9, 27, 36
8	50	2, 9, 20
9	50	3, 5, 9, 22, 32, 36
10	50	9, 14, 17, 22, 29

Anions		
2	70	3, 7, 9, 14, 19, 21, 28
3	70	5, 7, 9,11, 16, 26, 29
4	70	2, 7, 9, 12, 18, 25
5	60	2, 6, 9, 14, 19
6	60	5, 7, 9, 12, 15, 25

Similar magic numbers have been found previously for Cu^+^ upon electron ionization of neutral He nanodroplets doped with copper.^32^

In contrast, the next cluster size He_*m*_Cu_2_^+^ can attach only up to 80 He atoms and shows high ion intensity of the bare copper dimer followed by a substantial intensity drop for the first He atom attached. However, by *m* = 2, which is magic, the ion yield starts to increase in the high-pressure measurement, while in the low-pressure measurement it starts to increase only at *m* = 4, similar to the complexes containing a Cu^+^ metallic core. The magic numbers are *m* = 6, and *m* = 12, for the higher and lower pressures, respectively. We also observe additional less pronounced shell closures at *m* = 17 and 26.

He_*m*_Cu_3_^+^ complexes are more like He_*m*_Cu^+^, but even less He can be attached to the Cu_3_^+^ cluster ions (*m* < 70). Here again, we see very little ion signal from the bare cluster, which drops even more for the complex with one He attached. The most pronounced magic numbers obtained at high pressure are *m* = 3 and 6. The low-pressure measurement, in contrast, exhibits a shell closure at *m* = 18 and two magic numbers at *m* = 20 and 27, which are also in agreement with the high-pressure measurement.

Following the trend that we have seen for the previous cluster sizes, Cu_4_^+^ can attach even fewer He atoms with *m* < 60. Also, in this case, the signal is very low for the complexes with only a few He atoms attached. However, in the high-pressure data, we can immediately see a magic number at *m* = 2, followed by two less pronounced ones at *m* = 4 and 8. In both pressure regimes, we can find a shell closure at *m* = 24.

Attaching 10 He atoms less than Cu_4_^+^, the cluster size distribution of the He_*m*_Cu_5_^+^ complexes in Fig. 2.1.b shows a very prominent magic number at *m* = 4 at the high evaporation pressure, which is substantially reduced when the pressure is lowered. Another potentially magic number ion is *m* = 9. At high He pressure, a pronounced intensity drop follows this ion, and it has a slightly higher intensity than expected at low pressure.

Complexes with metallic cluster cores Cu_6_^+^ and Cu_7_^+^ only attach up to about 50 He atoms and exhibit similar trends. In both cases, there are no bare copper clusters visible and the distribution starts to grow with the first He attached. The addition of a second He atom makes the complex with *m* = 2 clearly magic. The He_*m*_Cu_6_^+^ complexes further show magic number ions at *m* = 4 and 9 in the low-pressure data. In contrast, He_*m*_Cu_7_^+^ not only show the magic ions with *m* = 7 and 9 at low pressure but also additional clear shell closures at *m* = 27 and 36 in the high-pressure data.

The maximum number of He atoms attached to the largest copper clusters investigated, containing *n* = 8, 9 and 10 copper atoms, is again about 50. The ion yields of all three copper core ions as a function of *m* exhibit similar distributions without pronounced magic numbers. When less He gas is added for the evaporation into the RF-hexapole (low pressure) no bare copper cluster ions are visible and the distributions start to grow only with the third He atom, while more evaporation gas enables the detection of the bare cluster ions and Cu_10_^+^ is even more abundant than HeCu_10_^+^. The most pronounced magic complex for He_*m*_Cu_8_^+^ is *m* = 20, however, in the high-pressure regime, we can also recognize *m* = 2 and 9 as magic. In the case of He_*m*_Cu_9_^+^ magic numbered ions are *m* = 9, 22, 32 and 36. However, less obvious ones can be identified from the second differences at *m* = 3 and 5. The ion yield distribution of He_*m*_Cu_10_^+^ exhibits stronger intensity anomalies than the ones of *n* = 8 and 9. Besides the earlier mentioned bare cluster ion, we also see in both pressure regimes a relatively strong magic ion at *m* = 14. Other possible magic numbers for this cluster size are *m* = 9, 17, 22 and 29. In general, due to the decreasing signal-to-noise ratio, the intensity of the peaks with *m* above 20 is less reliable, therefore the assignment of magic numbers in this range of He solvation becomes also less certain.

**Fig. 3:**
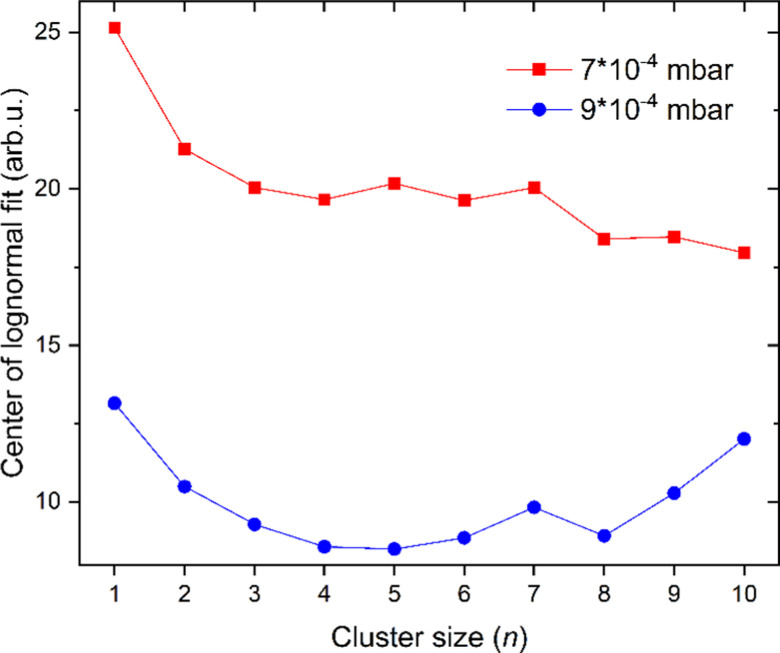
The cluster size distributions of He_*m*_Cu_*n*_^+^ that were measured at an evaporation pressure of 7 × 10^−4^ (red squares) and 9 × 10^−4^ mbar (blue circles) are fitted with lognormal distributions and the center of these fit functions is plotted against the cluster size *n*.

The amount of He atoms attached to cationic copper clusters decreases with increasing cluster size *n* as illustrated in Fig. 3. This effect can be mostly attributed to the decrease in partial charge since the positive charge becomes distributed among more copper atoms. Therefore, the binding energy between He and its neighboring copper atom(s) decreases. Previously it was already shown that the binding energy of Ar is decreasing with increasing copper cluster size,^19^ similar to the present case with He as a solvent. Moreover, the binding energy of He also typically decreases with additional He atoms adsorbed on cationic copper cluster ions. Binding of the first He reduces the overall charge on the copper core and therefore also polarization, similarly to the effects observed for the adsorption of Ar on Cu_*n*_^+^.^19^ We see a strong correlation between the magic numbers and the number of sites with the highest local charges.^19^

However, He can also be boiled off from the cluster ions due to additional energy which is provided by the absorption of black-body radiation. A black-body at room temperature (300 K) emits radiation in the IR with wavelength between 2 μm and up with a maximum emission of around 10 μm.^41^ In our previous work, we have shown that Cu_*n*_^+^ (*n* = 3–10) absorbs photons with energies between 33 and 100 μm.^18^ Therefore, it seems plausible that clusters can absorb energy from the black-body radiation emitted from the walls of the vacuum vessels.

Another explanation for the decreasing amount of He attached to larger copper cluster ions is the cross-section of the clusters for collisions with helium gas in the evaporation cell. Larger copper clusters are more likely hit by gas phase He atoms and the energy transferred to the cluster ions will finally lead to enhanced evaporation of He atoms attached.

**Fig. 4:**
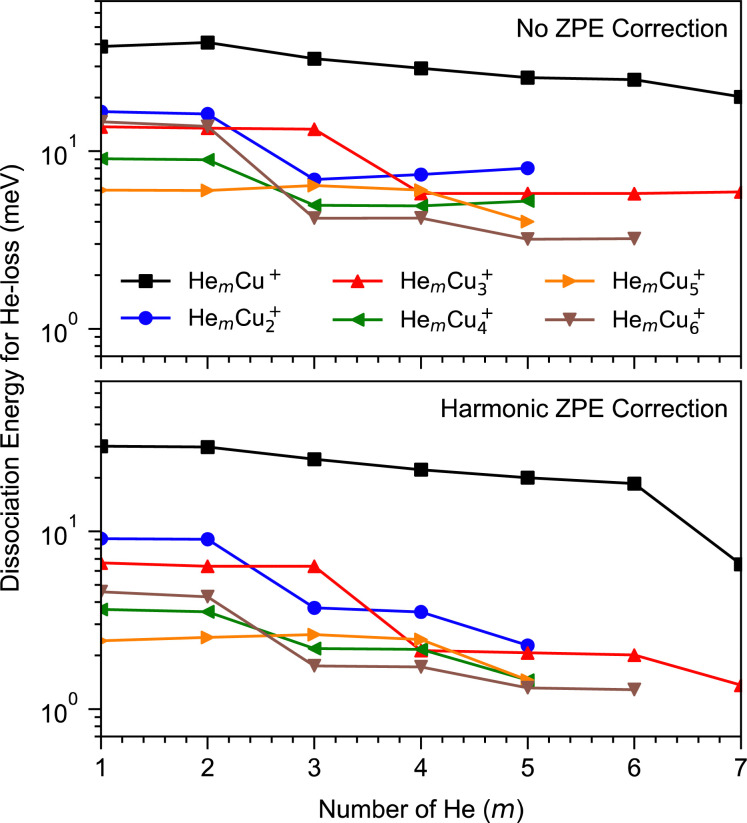
The calculated dissociation energy of He_*m*_Cu_*n*_^+^ clusters for different combinations of *m* and *n* when losing a single He atom. The top panel shows the calculated values without correcting for zero-point energies and the lower panel shows the same results when using a harmonic correction. The overall trend is that the binding energies of He to the Cu clusters decrease with increasing cluster size. Distinct drops in the binding energies, as more He atoms are added to a cluster, indicate (partial) solvation shell closures, the last of which in each series is only discernible when the ZPE correction is included. The positions of these steps are in good agreement with the magic numbers identified in the experimental data.

Our calculations have focused on the first few magic numbers present for cluster sizes up to six Cu atoms, in particular the trends seen for each cluster size. Beyond this, the number of available isomers becomes difficult to manage at the same time as the differences in interaction energies become very small between different positions in the weakly bound outer solvation layers. Calculated binding energies for the first few He atoms to different Cu_*n*_^+^ clusters are shown in Fig. 4. There is an overall trend that the average binding energies decrease with the increasing size of the copper clusters, *n*. This is attributed to the decrease of the average charge on the Cu atoms as the main attractive force acting on the He atoms is a charge-induced dipole effect. A similar trend was also observed earlier for Ar attachment on Cu_*n*_^+^.^19^ The most tightly bound He atoms are found for the lone Cu^+^ ion, where the first few He atoms are bound by approximately 40 meV each (without correcting for the zero-point energy, ZPE). Using a harmonic approximation to determine the ZPE, we see that this value decreases by one third. However, this reduction in binding energy is likely overestimated by this approximation. The largest effect that the zero-point correction has is for the CuHe_7_^+^ clusters. This occurs because the first six He atoms each occupy a local energy minimum around the Cu^+^ ion and interact only weakly with the other He atoms. However, when adding a seventh He, the coupling between some of the He atoms becomes relatively strong, resulting in a larger ZPE correction. Similar drops are clearly visible for the larger clusters as well at values of *m* that agree well with shell closures observed in the experiments.

**Fig. 5:**
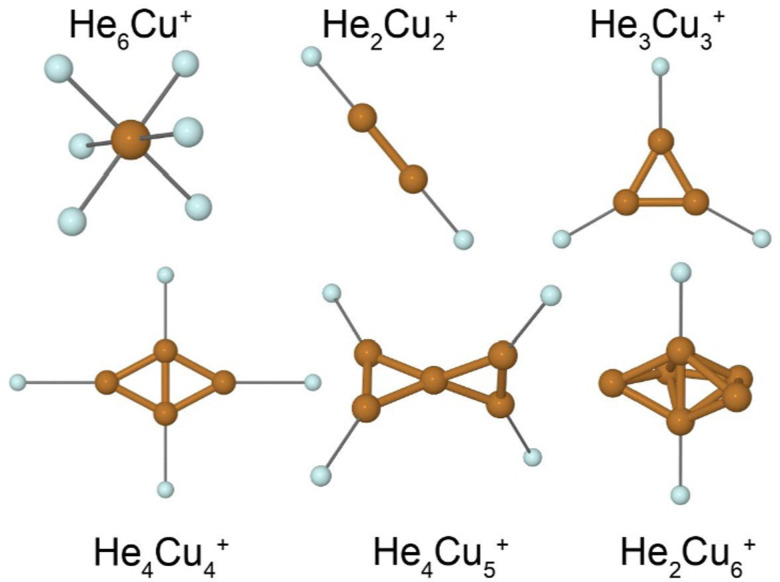
Proposed ground state structures of select He_*m*_Cu_*n*_^+^ clusters calculated at MP2/def2-TZVPD level of theory. Thin lines highlight the interaction between each He atom and its nearest neighboring Cu atom.

A few calculated structures are shown in Fig. 5. Our calculations predict that the cationic Cu clusters are two-dimensional for systems with less than 5 atoms and three-dimensional for systems with 5 or more atoms. The He_*m*_Cu^+^ systems show a distinct shell closure with *m* = 6 He atoms which gives the octahedral structure shown in Fig. 5. Clusters with *n* = 2, 3, or 4 Cu atoms all have the most tightly bound He atoms located in the plane of the metallic cluster. In all these cases, the symmetry of the He-tagged complex is equal to the symmetry of the bare metal clusters. In the case of *n* = 4, the first two He atoms (bound to the short axis of the rhombic structure of Cu_4_^+^ in Fig. 5) are more tightly bound than the subsequent two (along the long cluster axis). This is because the charge is not distributed equally amongst the atoms in the Cu_4_^+^ clusters. Instead, the two more closely positioned Cu atoms display a higher positive partial charge, attracting the He atoms, than the other two outer atoms.

The Cu_5_^+^ cluster is the smallest 3D structure that we identify. It displays a twisted-X geometry consisting of two triangles that are connected at a corner and which have their planes rotated by 90 degrees to each other. The four outer Cu atoms are identical, giving them all the same partial charge and thus the cluster can bind up to 4 He atoms with a nearly constant binding energy (see Fig. 5). This finding is in good agreement with the experimental results where *m* = 4 is strongly enhanced in the mass spectrum. Other possible isomers of Cu_5_^+^ are a trigonal bipyramid structure or planar structures such as a flat “X”. The prior lies approximately 70 meV higher in energy compared to the twisted-X geometry while the latter forms an unstable transition state in the electronic ground state, as do all attempts that we have made to produce a planar structure. The same relationships are also observed for isomers of He_*m*_Cu_5_^+^ clusters. The proposed structure of Cu_6_^+^ consists of a linear diatomic unit surrounded by a partial ring of the four remaining atoms. The separation between these atoms is consistent with the ring being able to occupy 5 or 6 atoms around the central axis. As is the case for the tetramer, the central pair of Cu atoms carries a higher partial charge than the surrounding atoms which attracts He atoms. The structure of He_2_Cu_6_^+^ in Fig. 5 shows the two most tightly bound positions for He atoms. Subsequent atoms may either share a position near the central pair of Cu atoms, or bind to one of the outer Cu atoms. All of these alternatives result in significantly lower binding energies than for the first 2 He atoms, again in good agreement with the experimental results.

### Anionic clusters

Similarly, we have studied the adsorption of He onto the Cu_*n*_^−^, with *n* = 2–8. Also, in this case, mass spectra at several pressure regimes and under different conditions have been measured. The full overview can be found in Fig. S5 (ESI†). Here, in Fig. 2.1.c, we show representative measurement performed for He_*m*_Cu_4_^−^ at evaporation pressures of 9.4 and 9.8 × 10^−4^ mbar called low and high pressure, respectively, with the corresponding second difference analysis in Fig. 2.2.c. For information on all measured structures see Fig. S3 and S4 (ESI†). The obtained data on magic numbers is summarized in Table 1.

Even from the first glance, it is clear, that the ion yield distributions for cations and anions as a function of *m* are different. First of all, for anions at every pressure and for every number of copper atoms *n* we see a strong presence of the bare copper cluster ion, which was nearly absent in the case of cations. Secondly, at nearly the same pressure 9 × 10^−4^ (cations) and 9.4 × 10^−4^ (anions), we see more He atoms attached to the anionic species. The maximum number of He attached for anions is around *m* = 70 for *n* = 2–4 and *m* = 60 for *n* = 5 and 6, while for cations it is below *m* = 50, except for *n* = 1. This can be readily explained by the larger number of He atoms per charge center for anions. Consequently, much higher pressure is required to reduce the amount of attached He.

The ion yields of anionic Cu clusters solvated with helium (He_*m*_Cu_*n*_^−^) exhibit quite a simple pattern as a function of the number of He atoms *m* attached. Most cluster sizes show magic number ions at *m* = 7 and 9, which, however, are not very reliable, since they may originate from isobaric impurities such as N_2_Cu_*n*_^+^ and (H_2_O)_2_Cu_*n*_^+^, respectively. These impurities, as discussed earlier, are affecting anions more severely, and even trace amounts in the residual gas or the helium gas used in the RF-hexapole for shrinking the He droplets lead to undesired contributions in the mass spectrum. Cu_4_^−^ and Cu_5_^−^ show a pronounced anomaly at *m* = 2, while Cu_3_^−^ and Cu_6_^−^ have a less pronounced one at *m* = 5. The full overview of magic numbers is given in the Table 1. There are also potentially other magic numbers at higher He decoration, but it is difficult to safely assign them due to the low signal intensity.

**Fig. 6:**
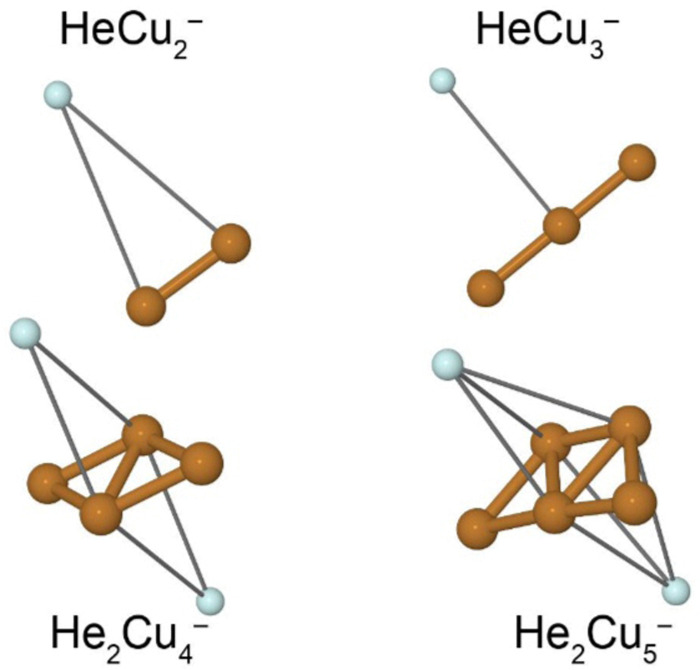
Proposed ground state structures of select He_*m*_Cu_*n*_^−^ clusters calculated at MP2/def2-TZVPD level of theory. Thin gray lines highlight the interaction between each He atom and its nearest neighboring Cu atom(s).

**Fig. 7:**
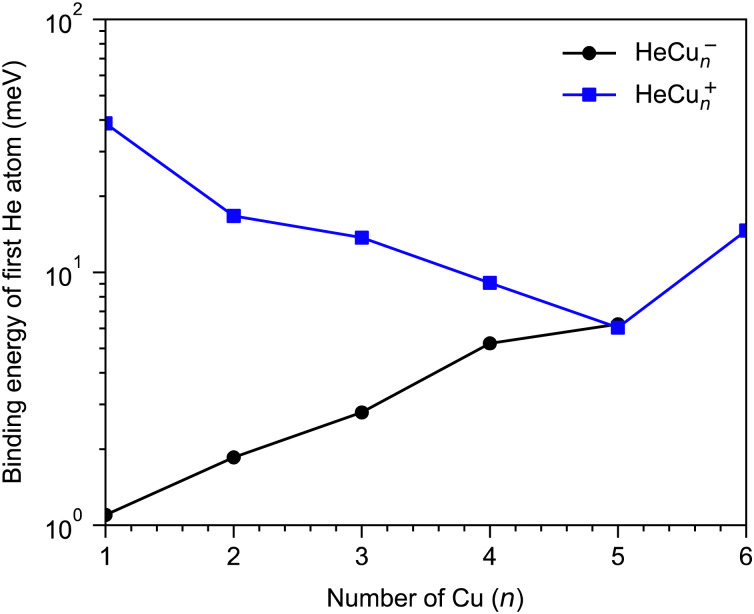
Calculated binding energy of the first He atom that binds to Cu_*n*_^+^ and Cu_*n*_^−^ clusters for different values of *n*, calculated at MP2/def2-TZVPD level of theory and with no ZPE correction. The overall trends are that the binding energies decrease with increasing cluster size for cations and increase with increasing size for anions until the two series converge.

A few example structures of anionic clusters are shown in Fig. 6. For the anionic clusters, the copper trimer is preferentially linear, unlike the case for the cations where a triangular structure was the energetically preferred structure. The calculations indicate that the excess negative charge is primarily localized near their centers, which is also where we find the He atoms preferentially bind to the cluster. This contrasts the cationic clusters where the He atoms preferentially occupied positions along the edges of the Cu_*n*_^+^ clusters. For the smallest HeCu^−^ complex, the binding energy is about 1 meV, more than an order of magnitude lower than the equivalent binding energy for cations. Unlike for cations, however, where the binding energies of He atoms generally decrease with increasing Cu_*n*_^+^ size, the binding energies of He to the Cu_*n*_^−^ clusters generally increase with increasing *n*. A comparison of the binding energy of the first He atom for cationic and anionic clusters is shown in Fig. 7. Here, we can clearly see that for the smallest clusters, cations bind He significantly stronger than their equivalent anions. As the cluster sizes increases though, the two curves converge as the role of the charge state diminishes. For large enough clusters, each curve is expected to converge on the behavior of neutral He_*m*_Cu_*n*_ clusters. The weaker interactions between the anionic clusters and the He atoms is due to their diffuse electronic structures which repel the rare gas atoms to greater distances than in the case of the cations.^42^ Due to this weak binding energies and complex potential energy surfaces, an extensive study of the structures of larger He_*m*_Cu_*n*_^−^ clusters lies beyond the scope of this present work. However, calculations on He_2_Cu_4_^−^ and He_2_Cu_5_^−^ clusters indicate that the pair of He atoms occupy positions opposite of each other above and below the plane of the copper cluster (see Fig. 6). Since the interaction between the He atoms is negligible at these positions, their binding energies will be nearly equal to that of the first He atom. These geometries likely explain the magic *m* = 2 numbers observed in the experiments for these cluster sizes.

## Conclusions

In this work, we have attempted to assign the structures of small ionic Cu clusters based on the stability of the complexes formed with He. All in all, the results presented in this work are in good agreement with theory^13–17^ and previous experiments.^18,20^ The only exception is Cu_5_^+^ for which both planar as well as trigonal bipyramid structures were previously assigned in the literature. The present results, however, indicate that Cu_5_^+^ has a twisted-X geometry. The difference in the shape can be attributed to the different methods used for cluster production, *i.e.*, condensation of the metal atoms around a charge centre in the He droplet instead of laser vaporization and gas aggregation. Another reason could be the utilization of a tag with a smaller mass, He instead of Ar. In their extensive computational search, the group of J. Robles came to the similar twisted-X geometry of Cu_5_^+^ as presented here.^17^ Another spectroscopic study with H_2_ adsorbed onto Cu_5_^+^ clusters suggests that the cluster structure might be fluctional since several low-energy structures are nearly isoenergetic and they all might contribute to the final spectrum.^43^ Similarly, the fluxional behaviour of Cu_5_ cluster was observed for the supported clusters.^44^ It should be noted, that structures formed in the He droplets might be unique for this method, since the cluster is forming at ultracold conditions around 0.4 K and every next atom attaches to the cold cluster core, unlike in other methods.

For all cationic copper clusters, He preferentially binds to individual surface atoms where also the highest charge density is found. In contrast, anionic copper clusters exhibit the highest negative charge density close to the center of the cluster. As in the cationic case, He preferentially binds to positions with the highest charge density.

Cluster growth in charged superfluid He droplets leads to the formation of ground state cluster structures, similar to that formed in a laser ablation source. The main advantage of the presently utilized method is its continuity and stability over long periods of time. He-tagging of cations and anions is highly beneficial for messenger-type spectroscopy with minimum perturbation of the cold ions due to the weak interaction of the ions with helium.^45^ Moreover, we have recently shown that the cluster production upon pickup into highly charged helium droplets can be size and charge-selective, leading to the increased signal of a desired cluster size.^24^ Therefore, the high yield of Cu_*n*_^+/−^ clusters formed by the present method can be used as a basis for further studies towards the reactivity of small copper clusters.

## Author contributions

OVL has done conceptualization, formal analysis, investigation, project administration, funding acquisition, visualisation and has written the original draft. MG has done formal analysis and visualisation. He also contributed to the writing of the original draft. JR was involved in the investigation and visualisation. SK and FZ were involved in the development of the methodology and investigation. MMD has reviewed and edited this manuscript. PS has done conceptualization, visualisation, funding acquisition, project administration and supervision. He has developed software, methodology and provided resources, as well as he has reviewed and edited this manuscript. All authors have read and agreed to the published version of the manuscript.

## Conflicts of interest

There are no conflicts to declare.

## Supplementary Material

CP-025-D2CP04569A-s001
