# Supplementary material for: Structure and formation of copper cluster ions in multiply charged He nanodroplets
Source: Phys Chem Chem Phys. 2023 Mar 14;25(12):8463–71. doi: 10.1039/d2cp04569a (PMC10032196; doi:10.1039/d2cp04569a)
Supplement: CP-025-D2CP04569A-s001 [file CP-025-D2CP04569A-s001.pdf]

Electronic Supplementary Material (ESI) for Physical Chemistry Chemical Physics.
This journal is © the Owner Societies 2023

Supporting information

Structure and formation of copper clusters in multiply charged superfluid He nanodroplets.

O.V. Lushchikova, M. Gatchell, J. Reichegger, S. Kollotzek, F. Zappa, M. Mahmoodi-Darian, P. Scheier

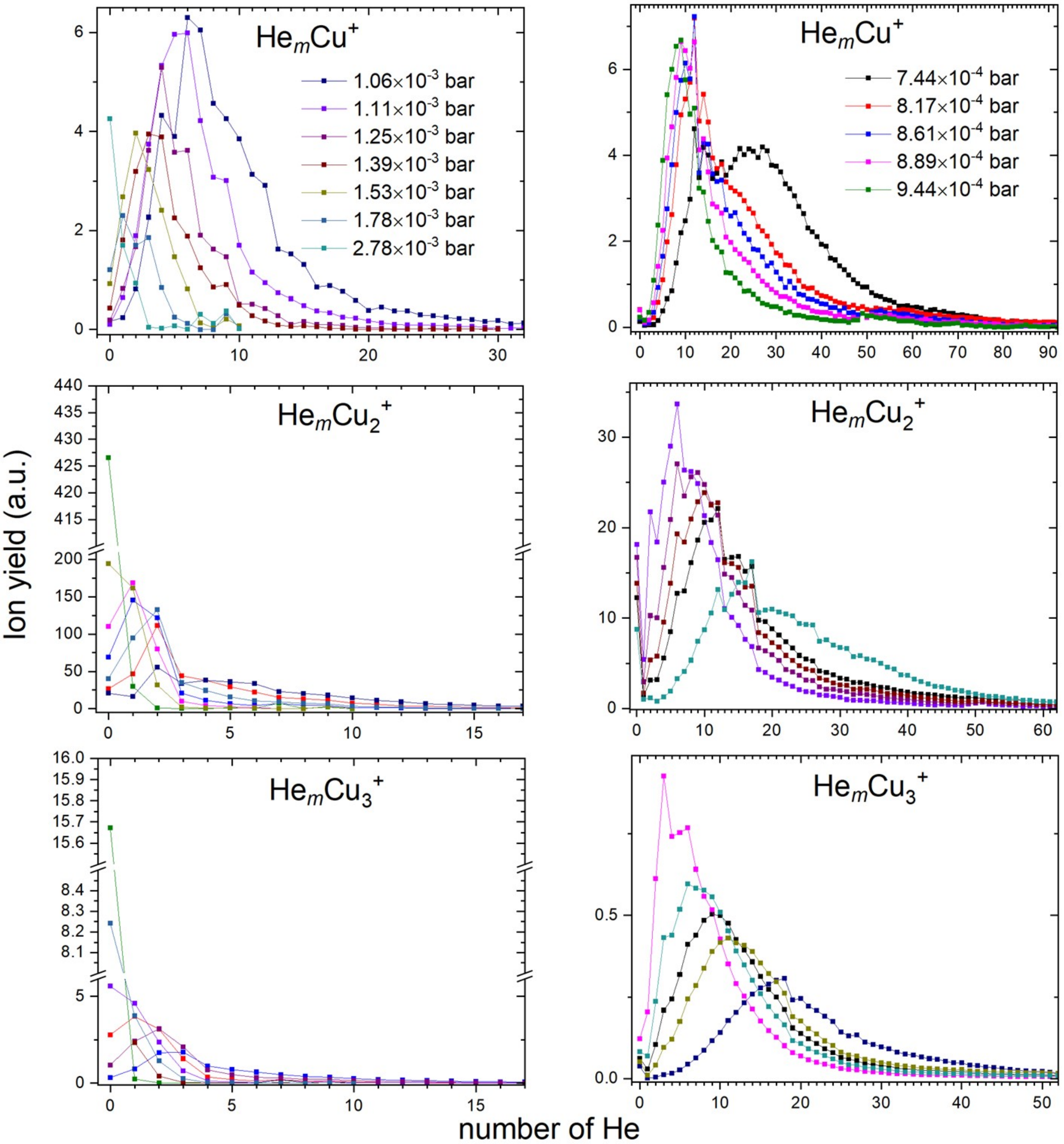

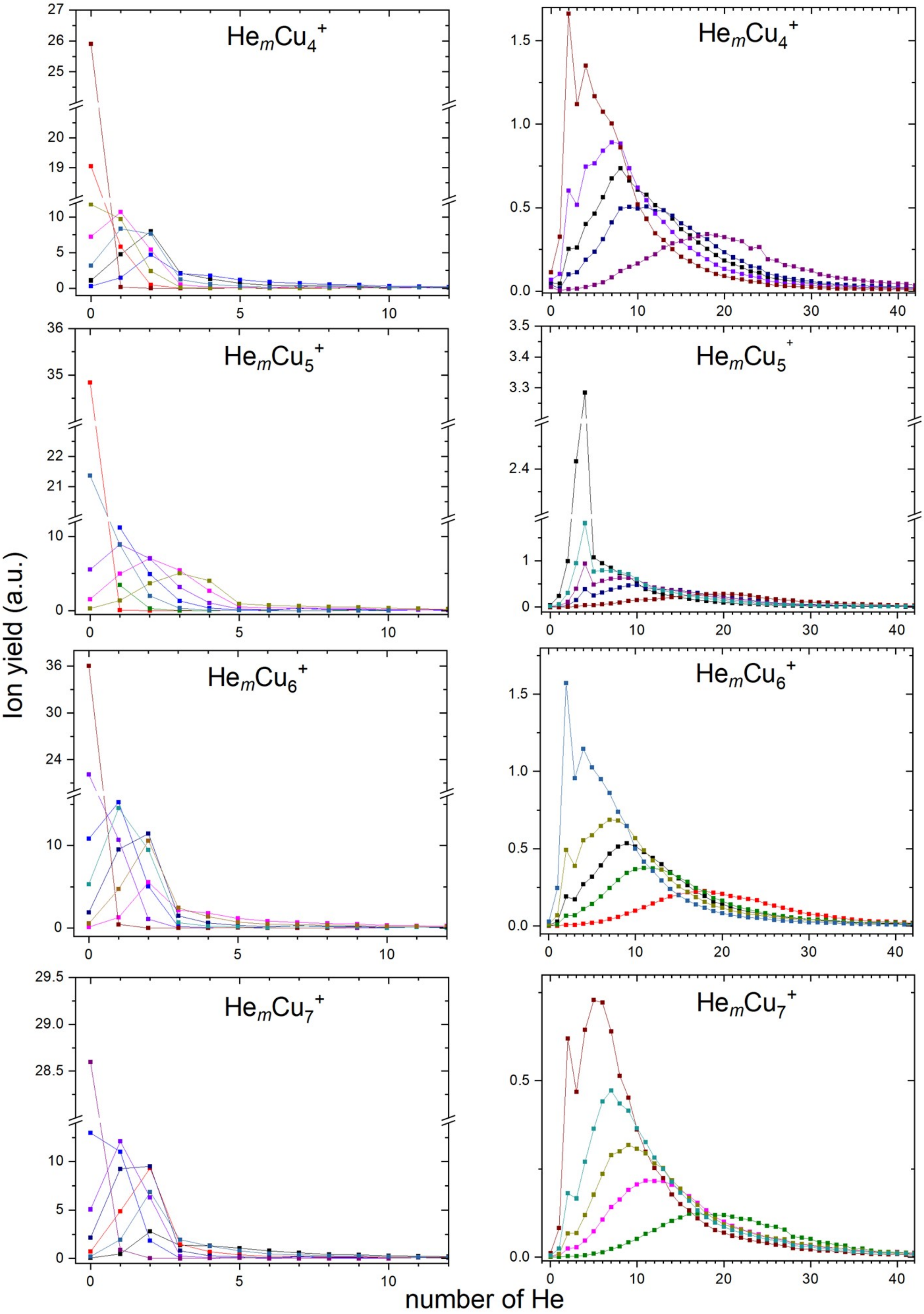

$He_mCu_4^+$
$He_mCu_4^+$
$He_mCu_5^+$
$He_mCu_5^+$
$He_mCu_6^+$
$He_mCu_6^+$
$He_mCu_7^+$
$He_mCu_7^+$
Ion yield (a.u.)
number of He

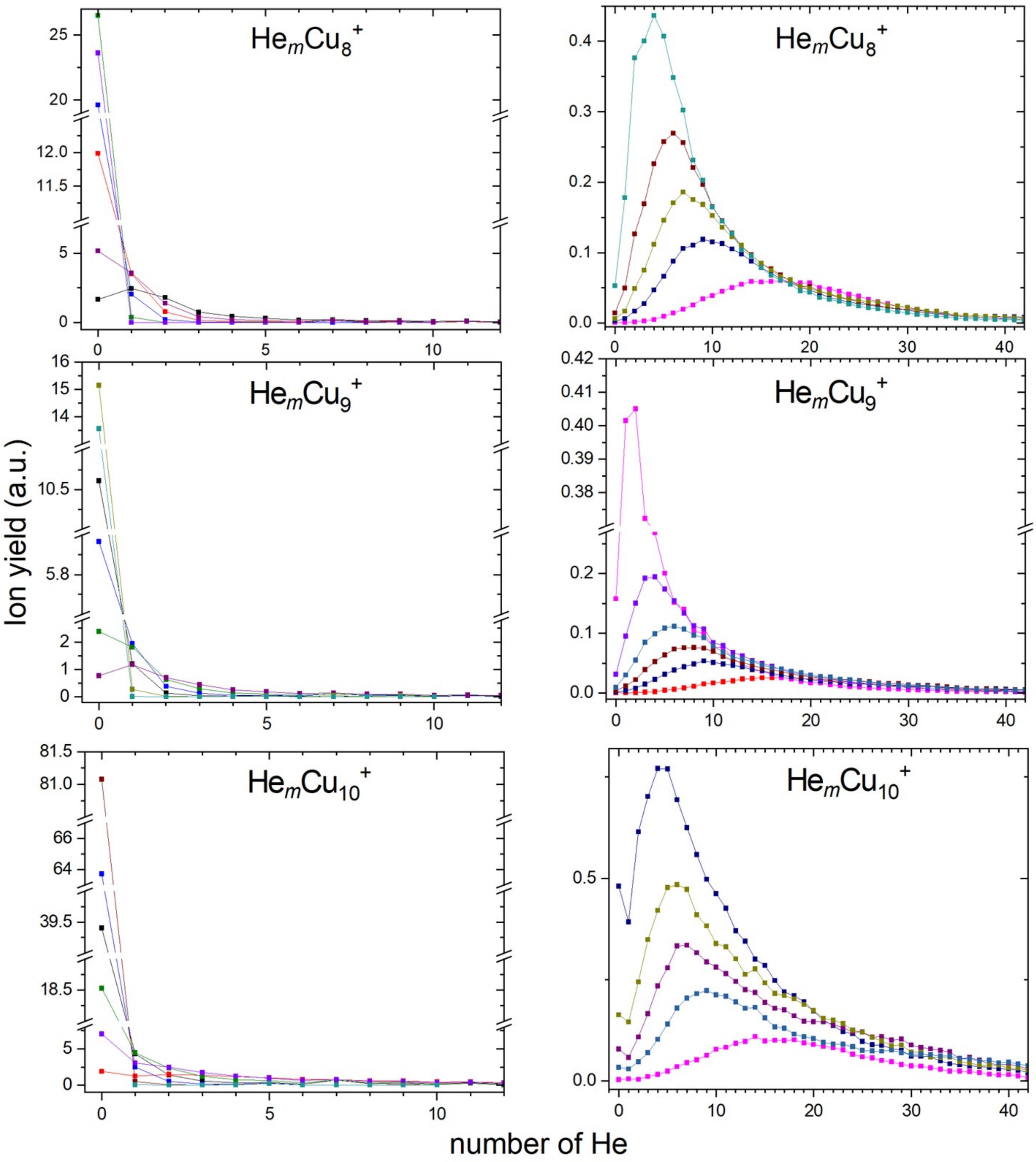

Figure S1: The cluster size distributions for He$_m$Cu$_n^+$ for 12 different pressures. Left graphs are high-pressure measurements from 3 to 1×10^{-3} mbar and right graphs are low-pressure measurements from 9 to 7×10^{-4} mbar. The distributions for each n are shown in separate plots and the symbols represent the ion yield of every m.

Table S1: Comparison of the experimental parameters for the original and reference measurements illustrated in figure S2.

Experimental parameters	Original	Reference
**He Pressure [bar]**	22	31
**Nozzle Temperature [K]**	9.5	9.8
**Ion Block Potential [V]**	285	365
**Deflector Vertical [V]**	10	6.5
**Deflector Horizontal [V]**	2.5	1.3
**Electron Energy [Ev]**	57	56
**Electron Current [Ua]**	350	290
**Float Voltage [V]**	-80	-30
**Deflect Voltage [V]**	-200	-200
**Float Z [V]**	15	-9
**U Z [V]**	6.5	1
**Float Y [V]**	4	10
**U Y [V]**	-33	-11
**Front Aperture [V]**	-300	0
**Oven Power [W]**	130	125
**Oven Temperature [C]**	779	750
**Evaporation Pressure [mbar]**	9×10^{-4}	9×10^{-4}

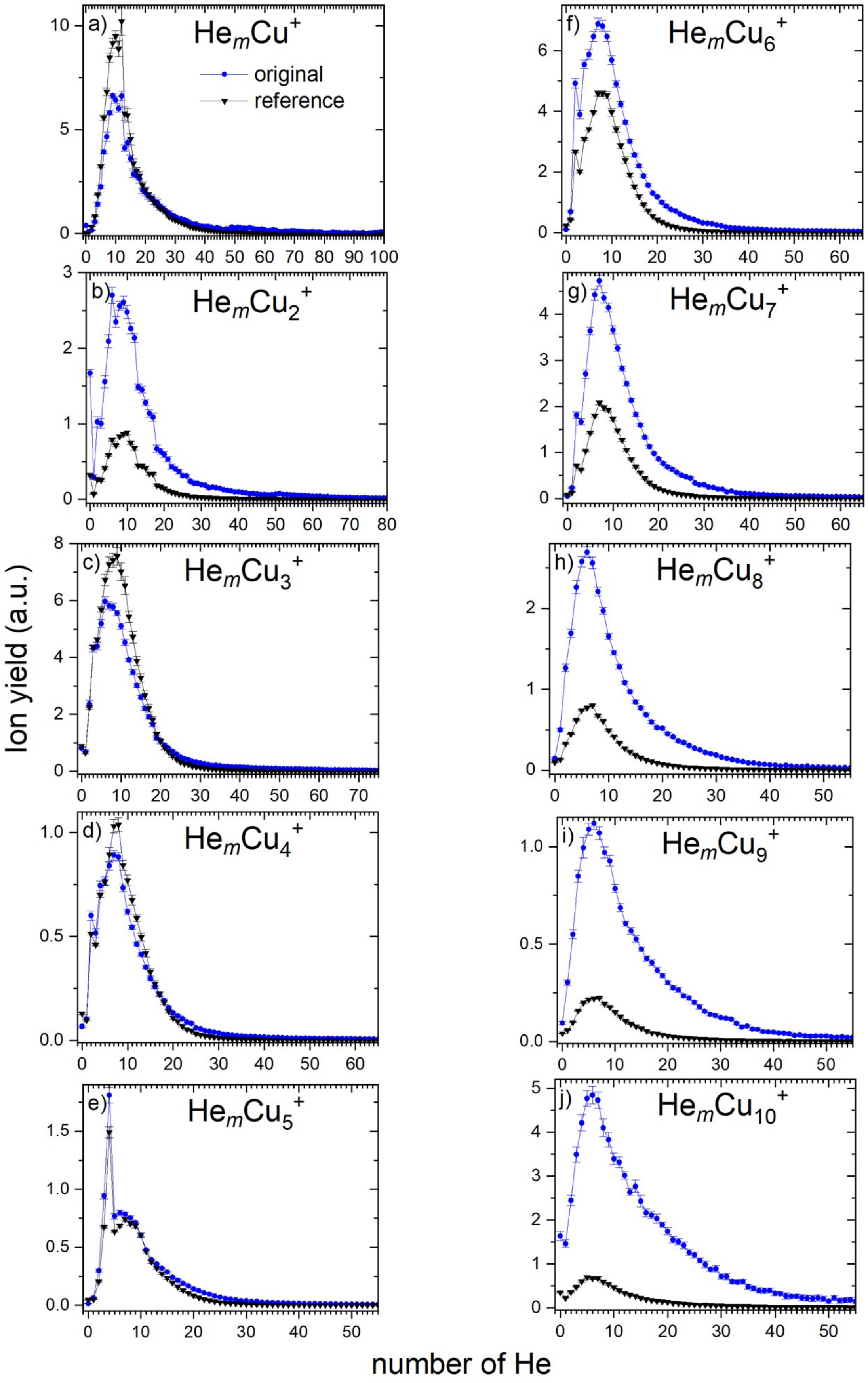

Figure S2: The cluster size distributions for Cu_n^+-He_m for original (blue circles) and reference (black triangles) measurements at 9×10^{-4} mbar. The distributions for each n are shown in separate plots. The symbols represent the ion yield of every m and are complemented with an error bar.

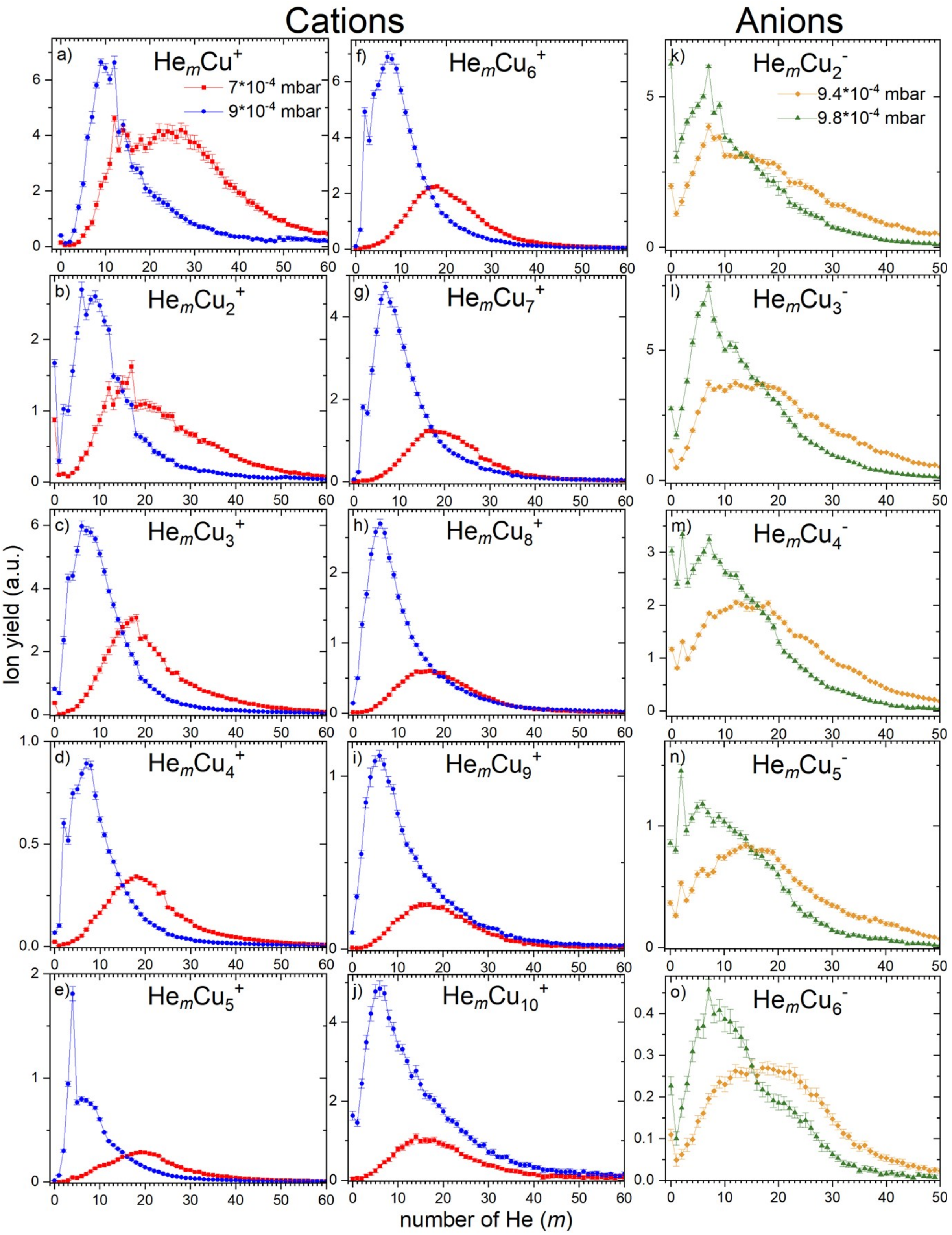

Figure S3: The cluster size distributions of $He_mCu_n^{+/-}$. Plots a-j show the cations measured at 7×10^{-4} (red squares) and 9×10^{-4} mbar (blue circles). Plots k-o show the distributions of anions measured at 9.4×10^{-4} (orange diamonds) and 9.8×10^{-4} (green triangles). The Hem distributions for each $Cu_n^{+/-}$ are shown in separate plots. The symbols represent the ion yield of every m and are complemented with an error bar.

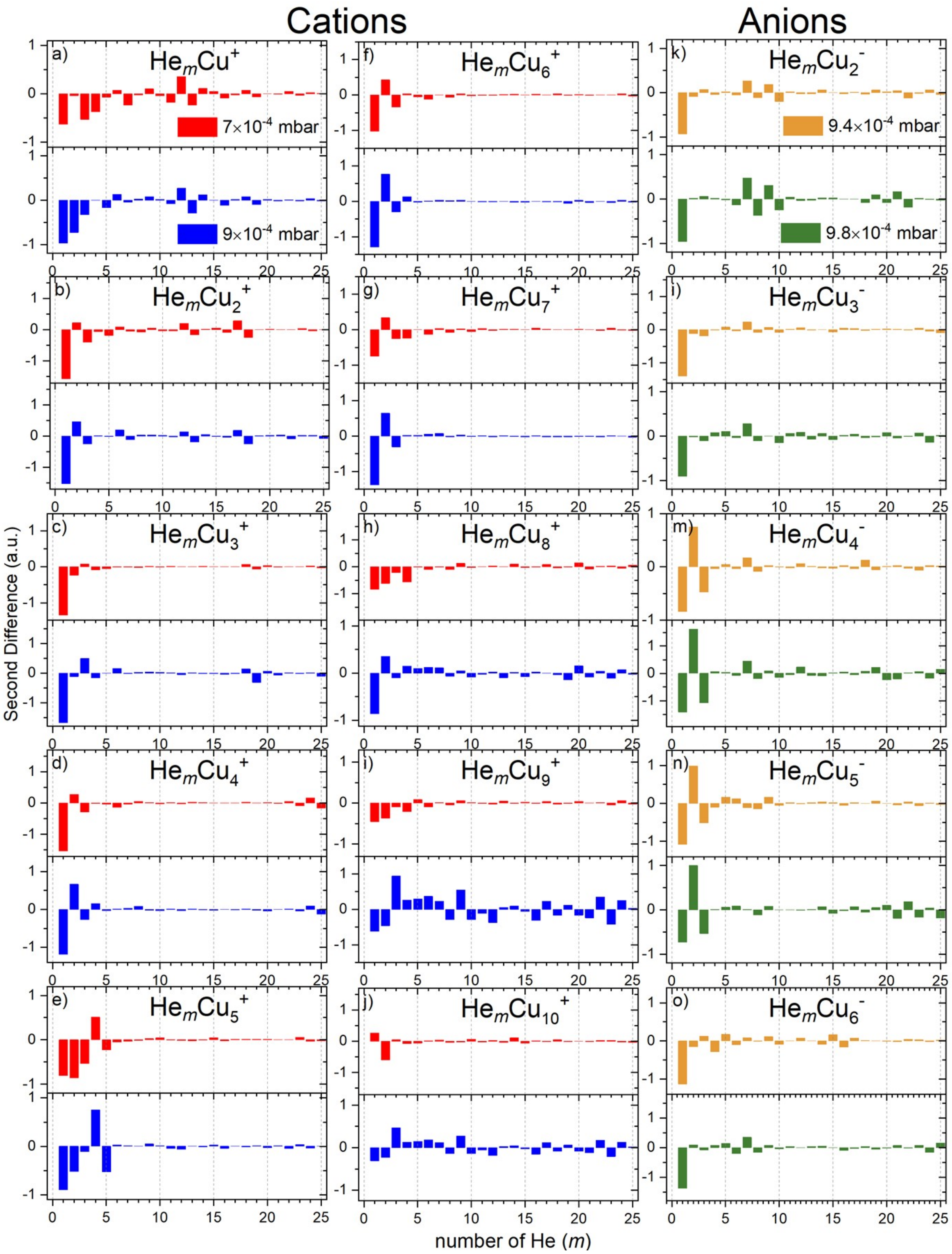

Figure S4: The second difference of the cluster size distributions of $He_mCu_n^{+/-}$. Plots a-j show the cations measured at 7×10^{-4} (red bars) and 9×10^{-4} mbar (blue bars). Plots k-o show the distributions of anions measured at 9.4×10^{-4} (orange bars) and 9.8×10^{-4} (green bars). The second differences of each Hem distribution for each Cu_n $^{+/-}$ are shown in separate plots.

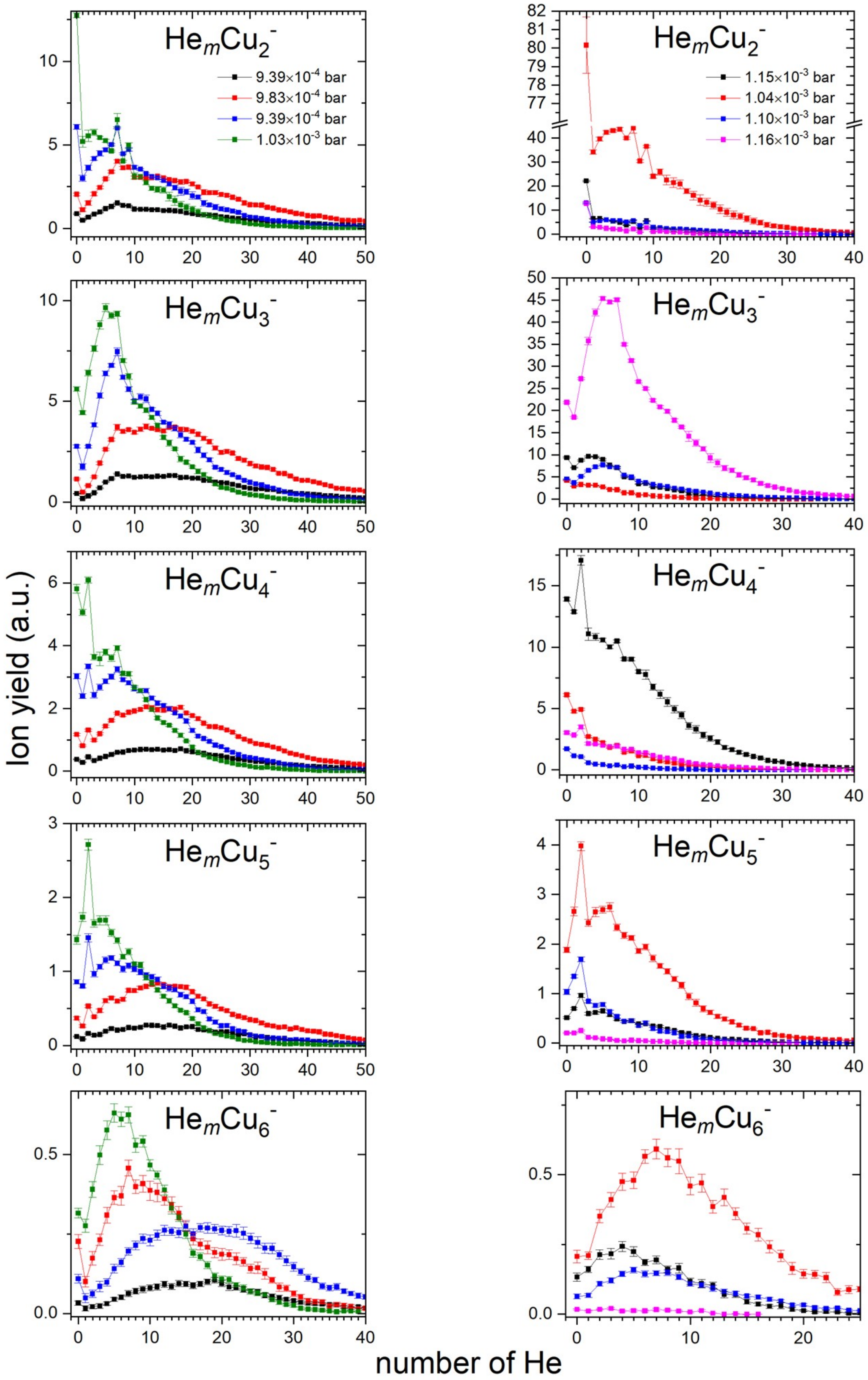

Figure S5: The cluster size distributions for $He_mCu_n^-$ measured at different pressures and conditions. The distributions for each *n* are shown in separate plots and the symbols represent the ion yield of every *m* accompanied by an error bar.

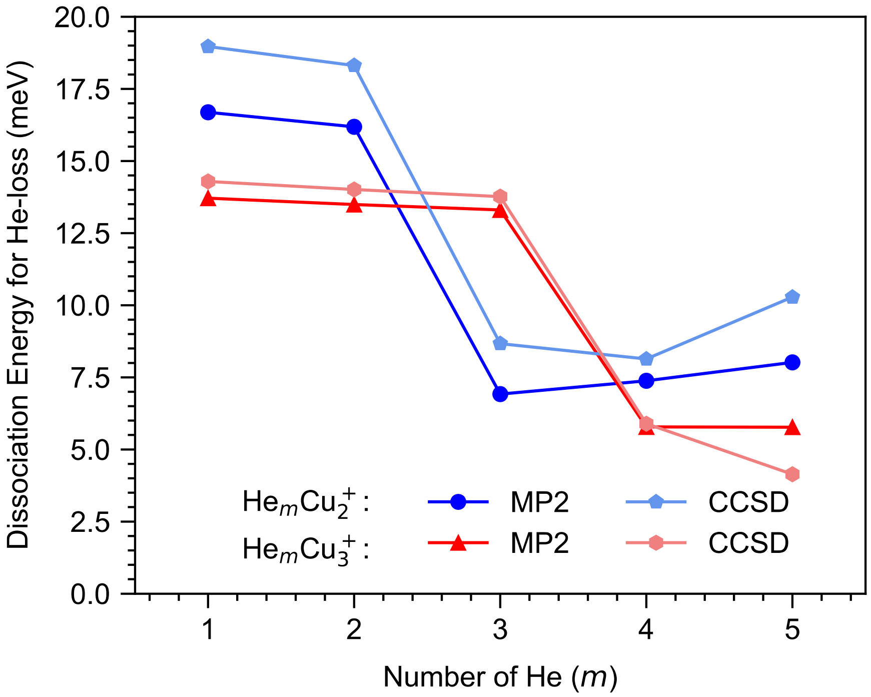

Figure S6: Comparison of binding energies of He determined for cationic Cu dimers and trimers from test calculations using the MP2 and CCSD methods, both with the def2-TZVPD basis set. The structure of each system was optimized on a counterpoise-corrected potential energy surface with the respective method. The results exclude any ZPE correction.

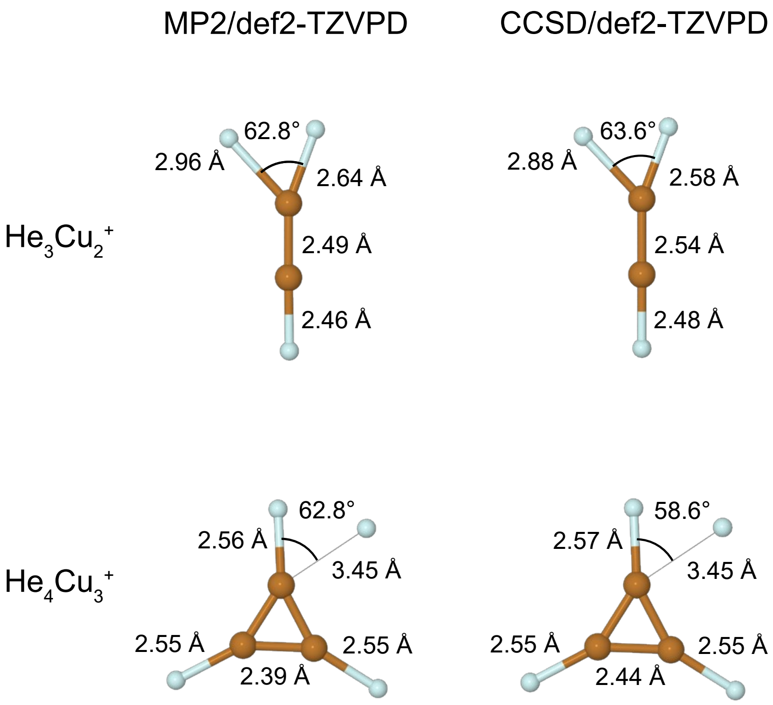

Figure S7: Select structures of $He_3Cu_2^+$ (top row) and $He_4Cu_3^+$ (bottom row) optimized at MP2/def2-TZVPD (left column) and CCSD/def2-TZVPD (right column) levels of theory. Bond lengths and angles vary slightly between the methods, but the geometries are otherwise very similar. This holds true for all structures we have tested with both methods.

Coordinates

The atomic coordinates obtained from our optimized clusters of $He_mCu_n^{+/-}$ are given below in order of increasing n and m starting with the cations. They are all obtained at MP2/def2-TZVPD level of theory using a counterpoise correction during each step of the optimization process. These are cartesian coordinates in units of Å (10^{-10} m).

Cations

$CuHe^+$

Cu	0.000000	0.000000	0.144227
He	0.000000	0.000000	-2.091291

$CuHe_2^+$

Cu	0.000000	0.000000	0.000000
He	0.000000	0.000000	2.194381
He	0.000000	0.000000	-2.194374

$CuHe_3^+$

Cu	0.000000	0.001446	-0.000000
He	1.955862	1.115412	0.000000
He	-0.000000	-2.251785	0.000000
He	-1.955862	1.115413	-0.000000

$CuHe_4^+$

Cu	0.012245	0.005690	0.016935
He	-0.580624	-2.038281	0.892598
He	2.048523	-0.588481	-0.875620
He	0.614959	2.043648	0.898590
He	-2.032618	0.605183	-0.851916

$CuHe_5^+$

Cu	-0.000000	0.001009	0.046101
He	-1.609796	1.598611	0.657099
He	1.679757	1.668650	0.177099
He	1.600062	-1.611131	0.651810
He	-1.670023	-1.681170	0.161810
He	-0.005000	0.010408	-2.316284

$CuHe_6^+$

Cu	0.000000	-0.000000	-0.000000
He	0.000000	-0.000000	2.389393
He	2.389393	0.000000	-0.000000
He	0.000000	-0.000000	-2.389393
He	-2.389393	-0.000000	-0.000000
He	-0.000000	2.389393	-0.000000
He	0.000000	-2.389393	-0.000000

$CuHe_7^+$

Cu	-0.030593	-0.004014	0.002573
He	-0.759794	-0.594356	2.228247
He	-0.598544	-1.612991	-1.726527
He	1.687693	0.464495	-1.652965
He	1.544336	1.265972	1.360818
He	1.526792	-1.832450	0.503950
He	-0.508104	2.286574	-0.648827
He	-2.448773	0.080955	-0.102005

Cu_2^+

Cu	-0.280128	0.530504	0.000000
Cu	2.222250	0.530504	0.000000

Cu_2He^+
Cu 0.000000 0.000000 -1.166886
Cu 0.000000 0.000000 1.333340
He 0.000000 0.000000 -3.641497

$Cu_2He_2^+$
Cu -0.000000 0.000000 -0.987887
Cu -0.000000 0.000000 1.510345
He -0.000000 0.000000 -3.475195
He -0.000000 0.000000 3.999848

$Cu_2He_3^+$
Cu 0.166451 1.179690 0.000000
Cu 0.035299 -1.309616 0.000000
He 1.205267 3.606498 0.000000
He -0.060417 -3.770307 0.000000
He -1.724095 3.463475 0.000000

$Cu_2He_4^+$
Cu -1.222537 0.372822 0.021639
Cu 1.230533 -0.094354 0.037381
He -3.568634 1.332713 -0.013532
He 3.615054 1.907345 0.000603
He -3.603226 -1.630043 -0.010436
He 3.578581 -1.053135 0.062848

$Cu_2He_5^+$
Cu 1.341951 -0.480441 0.056951
Cu -1.134602 -0.164751 0.028360
He -3.499826 -0.848586 -1.471340
He 3.747435 -1.267520 0.101108
He -3.520478 -0.737578 1.541801
He -3.209409 1.789664 -0.057863
He 3.589433 1.692666 -0.010213

Cu_3^+
Cu 2.105832 0.003579 0.000006
Cu 0.032048 1.195305 -0.000227
Cu 0.036193 -1.195314 0.000292

Cu_3He^+
Cu 0.748744 1.221172 -0.000262
Cu 0.784154 -1.168021 -0.000022
Cu -1.306711 -0.004086 0.000320
He -3.845619 -0.045380 0.000653

$Cu_3He_2^+$
Cu -0.017982 1.312636 -0.001269
Cu -1.185626 -0.771847 -0.000718
Cu 1.207612 -0.738327 0.000528
He 3.452469 -1.935228 -0.009447
He -3.379339 -2.058786 -0.002207

$Cu_3He_3^+$
Cu -0.205469 1.371007 0.023877
Cu -1.078948 -0.853404 0.025207
Cu 1.284089 -0.497725 0.029964
He 3.676483 -1.384043 0.024903
He -3.084120 -2.429698 0.021535
He -0.602924 3.890062 0.031641

$Cu_3He_4^+$
Cu 0.980397 -0.565401 0.000000
Cu -0.488302 1.319591 -0.000000
Cu -1.386265 -0.894954 0.000000
He 3.347581 -1.521807 0.000000
He -1.045521 3.823253 -0.000000

He 1.997562 3.712049 -0.000000
He -3.399683 -2.465940 0.000000

$Cu_3He_5^+$
Cu -1.218645 -0.730726 0.000000
Cu -0.411124 1.518116 -0.000000
Cu 1.132639 -0.305830 0.000000
He -3.166171 -2.385463 0.000000
He 1.977582 4.009967 -0.000000
He -1.068648 3.998040 -0.000000
He 2.105480 -3.614687 0.000000
He 3.611509 -0.969648 0.000000

$Cu_3He_6^+$
Cu 0.406749 1.318456 0.000000
Cu 0.938322 -1.010960 -0.000000
Cu -1.344799 -0.306611 0.000000
He -1.663418 4.082674 0.000000
He 1.358323 3.701393 0.000000
He 2.525580 -3.027553 -0.000000
He 4.366955 -0.601672 -0.000001
He -3.884210 -0.674092 0.000000
He -2.703501 -3.481637 0.000000

$Cu_3He_7^+$
Cu -1.108791 -0.873548 -0.021924
Cu -0.316013 1.376513 0.104940
Cu 1.239553 -0.434769 0.014841
He -0.838741 -4.310826 -0.207486
He -3.189212 -2.376952 -0.115174
He -0.579063 3.928318 0.242417
He -3.430982 2.863890 0.169300
He 3.531360 -1.636445 -0.042036
He 3.939763 1.022155 -1.471057
He 3.906135 0.805661 1.738887

Cu_4^+
Cu -0.005750 0.023427 0.052021
Cu 2.194756 -0.841958 0.751540
Cu 2.137413 0.814584 -0.878907
Cu 4.338256 -0.053182 -0.180586

Cu_4He^+
Cu -2.176203 -0.056487 -0.000455
Cu 0.001366 1.099799 0.001886
Cu -0.002217 -1.225797 -0.002903
Cu 2.175343 -0.063275 -0.000452
He -0.012599 -4.046925 -0.005216

$Cu_4He_2^+$
Cu 2.174938 0.007344 0.000240
Cu -0.003625 1.162838 -0.000772
Cu 0.003629 -1.162691 0.001387
Cu -2.174936 -0.006365 0.000238
He 0.018697 -3.991693 -0.002189
He -0.018845 3.992047 0.000084

$Cu_4He_3^+$
Cu -0.000000 -0.000000 2.202010
Cu -0.000000 1.162317 0.027242
Cu -0.000000 -1.162317 0.027242
Cu -0.000000 -0.000000 -2.148521
He 0.000000 -3.990401 0.026706
He 0.000000 3.990401 0.026706
He -0.000000 -0.000000 5.535041

$Cu_4He_4^+$

Cu	-0.000000	0.000000	2.175868
Cu	0.000000	1.161686	-0.000000
Cu	-0.000000	-1.161686	0.000000
Cu	-0.000000	0.000000	-2.175868
He	0.000000	-3.990561	0.000000
He	0.000000	3.990561	0.000000
He	-0.000000	0.000000	5.502269
He	0.000000	0.000000	-5.502269

$Cu_4He_5^+$

Cu	-2.174931	-0.048757	-0.133955
Cu	0.000336	1.103893	-0.279217
Cu	0.000110	-1.201910	0.014763
Cu	2.175630	-0.050387	-0.131249
He	-0.000647	-4.019241	0.417856
He	-0.001467	3.866834	-1.014186
He	-5.488728	-0.043025	-0.133957
He	5.489349	-0.046849	-0.127431
He	0.003745	3.626879	2.055745

Cu_5^+

Cu	0.000125	-0.001162	-0.000322
Cu	-2.171933	-0.849734	0.702150
Cu	2.191010	-0.790808	-0.713076
Cu	-2.098631	0.803576	-0.937554
Cu	2.079429	0.838127	0.948802

Cu_5He^+

Cu	-0.023038	-0.068538	-0.019218
Cu	2.174710	0.623701	0.769260
Cu	-2.231557	0.865601	-0.442913
Cu	2.036817	-0.617248	-1.197499
Cu	-2.071603	-1.106640	0.787203
He	-3.974151	3.012908	-1.696108

$Cu_5He_2^+$

Cu	0.071323	-0.000028	0.005341
Cu	-2.077073	-1.142967	-0.030850
Cu	2.206040	0.018477	-1.166508
Cu	-2.054002	1.185577	0.043466
Cu	2.214217	-0.058889	1.161618
He	-3.785555	-3.690988	-0.031925
He	-3.330630	3.939928	-0.052204

$Cu_5He_3^+$

Cu	0.024284	0.015720	-0.075300
Cu	2.181541	-1.070524	0.227501
Cu	-2.229145	0.012057	0.848586
Cu	2.112218	1.254268	0.099050
Cu	-1.971054	-0.122952	-1.462410
He	-3.672017	0.144194	3.514645
He	3.422863	-3.834721	0.426598
He	3.142916	4.116657	0.051052

$Cu_5He_4^+$

Cu	0.079841	0.011962	0.045668
Cu	2.230517	0.183226	-1.081084
Cu	-2.133104	-0.954006	-0.261662
Cu	2.193532	-0.455996	1.158386
Cu	-1.970831	1.282887	0.367071
He	-3.055645	4.036736	1.044066
He	-3.619726	-3.504184	-0.988147
He	3.610831	0.928073	-3.687752
He	3.326325	-1.345709	3.840739

$Cu_5He_5^+$

Cu	-0.132730	-0.092640	-0.028396

Cu 1.924732 -1.047420 -0.910931
Cu -2.276334 -0.777859 0.899053
Cu 2.075658 0.635512 0.691824
Cu -2.255031 0.818202 -0.796984
He 1.526301 -3.371006 -3.159136
He -3.362571 2.815084 -2.802555
He 3.383439 2.639551 2.567678
He 4.538627 -2.462825 -2.446791
He -3.422463 -2.660228 2.993945

Cu_6^+
Cu -0.011571 0.300584 1.200880
Cu -2.152806 1.280733 0.432098
Cu 2.120058 1.305794 0.437019
Cu -1.288790 -0.718238 -0.638466
Cu 1.279651 -0.704290 -0.637994
Cu -0.014687 1.378997 -0.819905

Cu_6He^+
Cu -0.000151 -0.353049 -1.216220
Cu -2.133923 -0.916657 -0.080705
Cu 2.133769 -0.916728 -0.081186
Cu -1.284750 1.358145 -0.010534
Cu 1.284727 1.358141 -0.010985
Cu 0.000050 -0.444060 1.073397
He -0.000105 -0.309281 -3.653632

$Cu_6He_2^+$
Cu -0.000231 -0.415479 -1.144418
Cu -2.132224 -0.940327 0.000628
Cu 2.131600 -0.940637 -0.000861
Cu -1.285842 1.336068 0.006775
Cu 1.285330 1.335915 0.006164
Cu -0.000259 -0.422514 1.146645
He -0.000886 -0.406890 -3.612992
He 0.000292 -0.407400 3.618622

$Cu_6He_3^+$
Cu 0.023061 -0.343673 -1.145494
Cu -2.111293 -0.855339 0.000422
Cu 2.152567 -0.875006 0.000089
Cu -1.254888 1.417101 0.000469
Cu 1.315976 1.405157 0.000344
Cu 0.023020 -0.344066 1.145538
He 0.022856 -0.328504 -3.617064
He 0.023044 -0.329397 3.616744
He 5.212488 -2.479746 0.001779

$Cu_6He_4^+$
Cu -0.002766 -0.346458 -1.145378
Cu -2.135731 -0.857569 0.000151
Cu 2.126810 -0.875138 -0.000108
Cu -1.280510 1.414929 -0.000583
Cu 1.289387 1.404376 -0.001021
Cu -0.001821 -0.344745 1.146629
He -0.003163 -0.329846 -3.617412
He -0.001319 -0.324260 3.618479
He 5.188686 -2.480997 0.001116
He -5.117039 -2.598120 0.002321

$Cu_6He_5^+$
Cu 0.005249 -0.418852 -1.151174
Cu -2.130885 -0.950335 -0.017851
Cu 2.132637 -0.968668 -0.011290
Cu -1.274835 1.322449 0.020808
Cu 1.296428 1.311318 0.025123
Cu 0.000895 -0.456639 1.139319

He -3.975359 3.883987 -0.032707
He 4.860831 -3.090281 -0.016999
He -4.678864 -3.304259 -0.024112
He -0.001563 -0.480141 3.612718
He 0.011464 -0.360142 -3.623947

$Cu_6He_6^+$
Cu 0.000572 -0.422681 -1.151771
Cu -2.135525 -0.954593 -0.016966
Cu 2.127681 -0.971418 -0.012225
Cu -1.281015 1.318233 0.020261
Cu 1.289567 1.307744 0.024308
Cu -0.004559 -0.460373 1.139574
He -3.983544 3.876568 -0.033843
He 3.973740 3.881852 -0.065451
He 4.857690 -3.092757 -0.017929
He -4.683029 -3.314208 -0.021934
He -0.008529 -0.479960 3.613830
He 0.004611 -0.360221 -3.625688

Anions

$CuHe^-$
Cu 0.000000 0.000000 0.401051
He 0.000000 0.000000 -5.920826

Cu_2^-
Cu 0.167581 1.196307 -0.000000
Cu 0.167581 -1.196096 0.000000

Cu_2He^-
Cu 0.679631 1.329588 -0.000000
Cu -1.169359 -0.188577 -0.000000
He -3.437612 4.454004 -0.000000

Cu_3^-
Cu 4.154495 -1.180706 0.000021
Cu 0.030161 1.180688 -0.000007
Cu 2.092329 -0.000009 0.000007

Cu_3He^-
Cu 1.351351 -0.041828 -1.143181
Cu -1.802552 0.055794 2.410433
Cu -0.227272 0.007035 0.632141
He 3.170033 -0.098121 3.648863

Cu_4^-
Cu 2.092331 -0.000022 -0.000007
Cu -0.000000 -1.197926 0.000007
Cu 0.000010 1.197963 0.000007
Cu -2.092341 -0.000016 -0.000007

Cu_4He^-
Cu -0.374801 0.005477 1.791581
Cu 0.613160 -1.207038 -0.046059
Cu 0.665991 1.189704 -0.035301
Cu 1.657488 -0.022881 -1.870968
He 4.220280 -0.094532 1.937419

Cu_5^-
Cu -0.000214 -0.793068 0.000681
Cu -1.192780 1.303138 -0.001472
Cu 2.350866 -0.904436 0.001163
Cu -2.351286 -0.904433 0.000952
Cu 1.192374 1.303135 -0.001323

Cu_5He^-

Cu	-0.000216	-0.793068	0.000684
Cu	-1.192735	1.303067	-0.001392
Cu	2.350775	-0.904359	0.001200
Cu	-2.351198	-0.904364	0.000912
Cu	1.192335	1.303059	-0.001403
He	0.006302	-0.020243	3.950447

$Cu_5He_2^-$

Cu	0.000108	-0.789060	0.002868
Cu	-1.192430	1.307057	-0.002939
Cu	2.351100	-0.900329	0.003662
Cu	-2.350873	-0.900377	0.003209
Cu	1.192640	1.307071	-0.002867
He	0.009200	-0.045562	-3.949596
He	0.006480	-0.009285	3.951265
